# Supplementary material for: Greedy gradient-free adaptive variational quantum algorithms on a noisy intermediate scale quantum computer
Source: Sci Rep. 2025 May 28;15:18689. doi: 10.1038/s41598-025-99962-1 (PMC12120033; doi:10.1038/s41598-025-99962-1)
Supplement: Supplementary file 1 — Supplementary Information. [file 41598_2025_99962_MOESM1_ESM.pdf]

Supplementary Information : Greedy Gradient-free Adaptive Variational Quantum Algorithms on a Noisy Intermediate Scale Quantum Computer

César Feniou[1,2], **Muhammad Hassan**[3], **Baptiste Claudon**[2], **Axel Courtat**[2], **Olivier Adjoua**[1], **Yvon Maday**[3], **and Jean-Philip Piquemal**[1,2,*]

[1]Sorbonne Université, Laboratoire de Chimie Théorique (UMR-7616-CNRS), F-75005 Paris, France
[2]Qubit Pharmaceuticals, Advanced Research Department, Paris, France
[3]Sorbonne Université, Université Paris Cité, CNRS, INRIA, Laboratoire Jacques-Louis Lions (LJLL), F-75005 Paris, France
[*]jean-philip.piquemal@sorbonne-universite.fr

ABSTRACT

Hybrid quantum-classical adaptive Variational Quantum Eigensolvers (VQE) hold the potential to outperform classical computing for simulating many-body quantum systems. However, practical implementations on current quantum processing units (QPUs) are challenging due to the noisy evaluation of a polynomially scaling number of observables, undertaken for operator selection and high-dimensional cost function optimization. We introduce an adaptive algorithm using analytic, gradient-free optimization, called Greedy Gradient-free Adaptive VQE (GGA-VQE). In addition to demonstrating the algorithm's improved resilience to statistical sampling noise in the computation of simple molecular ground states, we execute GGA-VQE on a 25-qubit error-mitigated QPU by computing the ground state of a 25-body Ising model. Although hardware noise on the QPU produces inaccurate energies, our implementation outputs a parameterized quantum circuit yielding a favorable ground-state approximation. We demonstrate this by retrieving the parameterized operators calculated on the QPU and evaluating the resulting ansatz wave-function via noiseless emulation (i.e., hybrid observable measurement).

1 Quantum circuits for qubit-excitation operators

For the sake of completeness, we present a few key quantum circuits used in the hardware experiments carried out for this study. The circuit for a single-qubit excitation is given in Figure 1 whereas the circuit for a double-qubit excitation is displayed in Figure 2. Both qubit excitations correspond to the qubit-excitation based (QEB) pool introduced in the main text Section 4.2, and, as explained in[1]. Note that although we used these quantum circuits for the QEB operators, we have since been made aware that more hardware-efficient circuits have been developed[2,3].

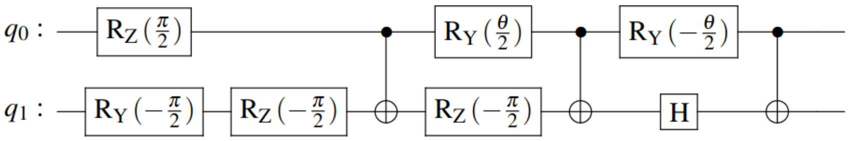

Figure 1. A quantum circuit performing a generic single-qubit evolution[1].

Remark: when displaying quantum circuits, H denotes the Hadamard gate and not a physical Hamiltonian.

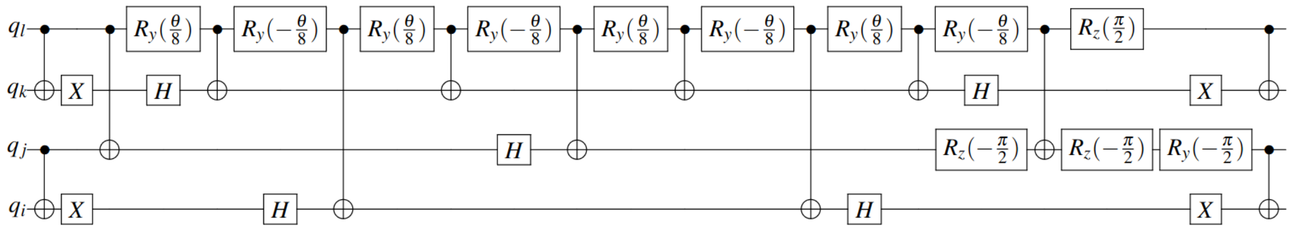

Figure 2. A quantum circuit performing a generic double-qubit evolution[1].

2 Periodicity of QEB operator pool and involutory property of hardware-efficient pools

Recall the definition of the qubit excitation-based (QEB) pool, A_{pqrs} denote a double-qubit excitation generator between the pairs of qubits (p,q) and (r,s),

$$A_{pqrs} = \frac{1}{8}\left(X_rY_sX_pX_q + Y_rX_sX_pX_q + Y_rY_sY_pX_q + Y_rY_sX_pY_q - X_rX_sY_pX_q - X_rX_sX_pY_q - Y_rX_sY_pY_q - X_rY_sY_pY_q\right).$$

A direct calculation shows that A_{pqrs} can be written in the equivalent form

$$A_{pqrs} = i(Q_p^\dagger Q_q^\dagger Q_r Q_s - Q_r^\dagger Q_s^\dagger Q_p Q_q),$$

where for any qubit index a, we define $Q_a = \frac{1}{2}(X_a + iY_a)$.

Using this representation, we can easily show that $A_{pqrs}\,|1_p1_q0_r0_s\rangle = i\,|0_p0_q1_r1_s\rangle$, $A_{pqrs}\,|0_p0_q1_r1_s\rangle = -i\,|1_p1_q0_r0_s\rangle$ while the action of A_{qprs} on all other states is zero. Consequently, the subspace spanned by $e_1 \equiv |1_p1_q0_r0_s\rangle$ and $e_2 \equiv |0_p0_q1_r1_s\rangle$ is an invariant subspace of A_{pqrs}, and in the basis $\{e_1, e_2\}$ of this invariant subspace, A_{pqrs} has the representation

$$A_{pqrs} = \begin{pmatrix} 0 & -\imath \\ \imath & 0 \end{pmatrix},$$

which is the well-known Y Pauli matrix. We thus conclude that A_{pqrs} has eigenvalues $0, \pm 1$ and satisfies $A_{pqrs}^3 = A_{pqrs}$.

A similar demonstration can be carried out for single-qubit generators from the QEB pool and generators from the qubit hardware efficient and minimal hardware efficient pools, which shows that these generators are involutory, i.e., they satisfy $B^2 = I$. For the sake of brevity, we do not provide a detailed argument.

The above observation motivates further investigation and leads to the following result.

Theorem 1. *Let H denote an N-qubit Hamiltonian and let $\mathbb{P}$ denote any of the operator pools introduced in Section 4.2. Then define, for any N-qubit wave-function $|\phi\rangle$, any Hermitian generator $B \in \mathbb{P}$ and any $\theta \in [-\pi, \pi)$ the landscape function*

$$\mathscr{L}(B, \theta, |\phi\rangle) = \langle \phi\,|\exp(\imath\theta B) H \exp(-\imath\theta B)|\,\phi\rangle.$$

Then it holds that

$$\mathscr{L}(B,\theta,|\phi\rangle) = \begin{cases} \langle\phi|H|\phi\rangle + \big(\cos(\theta)-1\big)\big(\langle\phi|\{H,B^2\}|\phi\rangle - 2\,\langle\phi|BHB|\phi\rangle\big) & \text{if } B^3 = B, \\ +\big(1-\cos(\theta)\big)^2\big(\langle\phi|B^2HB^2|\phi\rangle - \langle\phi|BHB|\phi\rangle\big) & \\ +\sin(\theta)(\cos(\theta)-1)\,\langle\phi|\imath B[H,B]B|\phi\rangle & \\ +\sin(\theta)\,\langle\phi|\imath[B,H]|\phi\rangle & \\ & \\ \cos^2(\theta)\,\langle\phi|H|\phi\rangle + \dfrac{\sin(2\theta)}{2}\,\langle\phi|\imath[B,H]|\phi\rangle & \text{if } B^2 = I. \\ +\sin^2(\theta)\,\langle\phi|BHB|\phi\rangle, & \end{cases}$$

where $\{\cdot,\cdot\}$ and $[\cdot,\cdot]$ denote the anti-commutator and commutator respectively.

Proof. We consider first the case $B^3 = B$. For such a Hermitian generator, we can use the Taylor series expansion of the exponential to deduce that

$$\begin{aligned}\exp(-\imath\theta B) &= \sum_{k=0}^{\infty}\frac{(-i\theta B)^{2k}}{(2k)!} + \sum_{k=0}^{\infty}\frac{(-i\theta B)^{2k+1}}{(2k+1)!} \\ &= I + (\cos(\theta)-1)B^2 - \imath\sin(\theta)B.\end{aligned} \tag{1}$$

Plugging in the expression (1) into the definition of the landscape function $\mathscr{L}(B,\theta,|\phi\rangle)$ now yields the desired result. The case $B^2 = I$ is simply a special case. ☐

3 Analytical expressions of GGA-VQE objective functions for the Ising Hamiltonian

Throughout this section, we use the setting and notation of Section (4.4) from the main text. Our goal now is to demonstrate that for the Ising Hamiltonian defined through Equation (17) and the minimal hardware-efficient pool $\mathbb{P}$ given by equation (18), the objective function $\mathscr{L}(B,\theta,|\Psi^{(m-1)}\rangle)$ has the following simple structure:

$$\mathscr{L}(B,\theta,|\Psi^{(m-1)}\rangle) = \begin{cases} \left\langle\Psi^{(m-1)}\middle|H\middle|\Psi^{(m-1)}\right\rangle & \text{if } B = Y_p, \\ +\sin(2\theta)\left\langle\Psi^{(m-1)}\middle|hZ_p - J(X_pZ_{p+1}+Z_{p-1}X_p\delta_{p>0})\middle|\Psi^{(m-1)}\right\rangle & \\ -2\sin^2(\theta)\left\langle\Psi^{(m-1)}\middle|hX_p + J(Z_pZ_{p+1}+Z_{p-1}Z_p\delta_{p>0})\middle|\Psi^{(m-1)}\right\rangle. & \\ & \\ \left\langle\Psi^{(m-1)}\middle|H\middle|\Psi^{(m-1)}\right\rangle & \text{if } B = Z_pY_{p+1}. \\ +\sin(2\theta)\left\langle\Psi^{(m-1)}\middle|h(Z_pZ_{p+1}-Y_pY_{p+1})\middle|\Psi^{(m-1)}\right\rangle & \\ -\sin(2\theta)\left\langle\Psi^{(m-1)}\middle|J(X_{p+1}+Z_pX_{p+1}Z_{p+2}\delta_{p+2<n})\middle|\Psi^{(m-1)}\right\rangle & \\ -2\sin^2(\theta)\left\langle\Psi^{(m-1)}\middle|hX_p + hX_{p+1} + JZ_pZ_{p+1} + JZ_{p+1}Z_{p+2}\delta_{p+2<n}\middle|\Psi^{(m-1)}\right\rangle. & \end{cases} \tag{2}$$

To show that Equation (2) indeed holds, we first recall the definition of the objective function $\mathscr{L}(B,\theta,|\Psi^{(m-1)}\rangle)$ which is given by

$$\mathscr{L}(B,\theta,|\Psi^{(m-1)}\rangle) = \langle\Psi^{(m-1)}|\exp(\imath\theta B)H\exp(-\imath\theta B)|\Psi^{(m-1)}\rangle, \tag{3}$$

where $B\in\mathbb{P}$ is any Hermitian generator from the minimal hardware-efficient operator pool, the parameter $\theta\in[-\pi,\pi)$, and $|\Psi^{(m-1)}\rangle$ denotes the previous ansatz wave-function.

Next, we recall that the involutory property of the Hermitian generators from the minimal hardware-efficient pool yields the following simplification of Equation (3):

$$\mathscr{L}(B,\theta,|\Psi^{(m-1)}\rangle) = \cos^2(\theta)\langle\phi|H|\phi\rangle + \frac{\sin(2\theta)}{2}\langle\phi|\imath[B,H]|\phi\rangle + \sin^2(\theta)\langle\phi|BHB|\phi\rangle. \tag{4}$$

Consequently, in order to arrive at Equation (2), we have to simplify each term involving the Ising Hamiltonian and minimal hardware-efficient generator B appearing in Equation (4).

To do so, recall that we denote the total number of qubits (i.e., the size of the quantum register) by $N\in\mathbb{N}$, and fix an index $p\in\{0,\dots,N-2\}$. Using now the commutation relations of the Pauli matrices, a direct calculation reveals that

$$[Y_p,H] = -2h\imath Z_p + 2\imath J(X_pZ_{p+1}+Z_{p-1}X_p\delta_{p>0})$$

and

$$[Z_pY_{p+1},H] = 2\imath h(Y_pY_{p+1}-Z_pZ_{p+1}) + 2\imath J(X_{p+1}+Z_pX_{p+1}Z_{p+2}\delta_{p<N-2}). \tag{5}$$

A similar calculation utilizing once again the commutation relations of the Pauli matrices further yields that

$$Y_pHY_p = H - 2hX_p - 2J(Z_pZ_{p+1}+Z_{p-1}Z_j\delta_{p>0}),$$

and

$$ZZ_{p+1}HZ_pZ_{p+1} = \sum_{q=0}^{N-1} Z_pZ_{p+1}X_qZ_pZ_{p+1} = H - 2h(X_p + X_{p+1}). \tag{6}$$

The result now follows by plugging in Equations (5) and (6) into Equation (4).

4 Reducing the computational complexity of the energy sorting algorithm for general spin chains

As demonstrated in Section 4.4, the specific structure of transverse-field Ising Hamiltonian leads to a huge reduction in the computational cost of the energy sorting step of the GGA-VQE algorithm. Indeed, while the energy sorting step a priori requires $\mathscr{O}(M)$ measurements for a general system Hamiltonian and an operator pool of size M, the number of required measurements reduces to just five in the case of the one-dimensional transverse field Hamiltonian. The goal of this section is to briefly describe similar reductions in the computational complexity of the energy sorting algorithm for Ising spin-chain Hamiltonians with local magnetic fields and couplings in all three spatial directions, i.e., Hamiltonians of the form

$$H = \sum_{k=0}^{N-1} h_k^x X_k + \sum_{k=0}^{N-1} h_k^z Z_k + \sum_{k=0}^{N-2} J_k^x X_k X_{k+1} + \sum_{k=0}^{N-2} J_k^y Y_k Y_{k+1} + \sum_{k=0}^{N-2} J_k^z Z_k Z_{k+1}. \tag{7}$$

Here, h_k^x and h_k^z denote constants that model the intensity of the magnetic field along the x and z directions while J_k^x, J_k^y and J_k^z are constants that model the strength of the nearest-neighbor interactions in the x, y, and z directions respectively.

Tables 1 and 2 list the terms of interest that appear in the one-dimensional GGA-VQE landscape functions that are used to perform the energy sorting step. Comparing the terms that appear in Tables 1 and 2 with the simpler expressions for the transverse-field Ising Hamiltonian from Section 4.4, we see that the only new terms that arise are of the $Z_{p-2}Z_{p-1}X_p$ and $Y_{p-1}X_p$. As before, we can simultaneously measure such operators acting on a disjoint set of qubits– a process that will require an additional five quantum circuits at each step. Consequently, applying the GGA-VQE algorithm to general Ising Hamiltonians of the form (7) will require constructing and measuring at most ten quantum circuits, irrespective of the number of qubits and the size of the minimal operator pool.

$\mathbf{H}$	$\sum \mathbf{h_k X_k}$	$\sum \mathbf{h_k Y_k}$	$\sum \mathbf{h_k Z_k}$
$[\mathbf{Y_i}, \mathbf{H}]$	$-2ih_iZ_i$	0	$2ih_iX_i$
$\mathbf{Y_i H Y_i}$	$H - 2h_iX_i$	H	$H - 2Z_i$
$[\mathbf{Z_i Y_{i+1}}, \mathbf{H}]$	$-2ih_iZ_iZ_{i+1} + 2ih_{i+1}Y_iY_{i+1}$	$-2ih_iX_iY_{i+1}$	$2ih_{i+1}Z_iX_{i+1}$
$\mathbf{Z_i Y_{i+1} H Z_i Y_{i+1}}$	$H - 2h_{i+1}X_{k+1} - 2h_iX_i$	$H - 2h_{i+1}Y_{i+1}$	$H - 2h_iZ_i - 2h_{i+1}Z_{i+1}$

Table 1. Commutators involving generators from the minimal operator pool and the local magnetic field terms.

$\mathbf{H}$	$\sum \mathbf{J_k X_k X_{k+1}}$	$\sum \mathbf{J_k Y_k Y_{k+1}}$	$\sum \mathbf{J_k Z_k Z_{k+1}}$
$[\mathbf{Y_i}, \mathbf{H}]$	$-2iJ_iZ_iX_{i+1} - 2iJ_{i+1}X_iZ_{i+1}$	0	$2iJ_iX_iZ_{i+1} + 2iJ_{i-1}Z_{i-1}X_i$
$\mathbf{Y_i H Y_i}$	$H - 2J_iX_iX_{i+1} - 2J_{i+1}X_{i-1}X_i$	H	$H - 2J_iZ_iZ_{i+1} - 2J_{i-1}Z_{i-1}Z_i$
$[\mathbf{Z_i Y_{i+1}}, \mathbf{H}]$	$-2iJ_{i+1}Z_iZ_{i+1}X_{i+2} - 2iJ_{i-1}X_{i-1}Y_iY_{i+1}$	$-2iJ_iX_i - 2iJ_{i-1}Y_{i-1}X_iY_{i+1}$	$2iJ_iX_{i+1} + 2iJ_{i+1}Z_iX_{i+1}Z_{i+2}$
$\mathbf{Z_i Y_{i+1} H Z_i Y_{i+1}}$	$H - 2J_{i-1}X_{i-1}X_i$	$H - 2J_iY_iY_{i+1} - 2J_{i-1}Y_{i-1}Y_i$	$H - 2J_{i+1}Z_{i+1}Z_{i+2} - 2J_iZ_iZ_{i+1}$

Table 2. Commutators involving generators from the minimal operator pool and the interaction terms in each direction.

Finally, let us remark that we expect similar but likely less drastic simplifications to also hold for Hamiltonians arising from other physical models.

5 Multi-dimensional analytical landscape functions and post-processing of GGA-VQE

Let us consider an adaptive procedure in which d unitary operators, constructed using d Hermitian generators from a given operator pool $\mathbb{P}$ are to be appended to the current ansatz wave-function $|\Psi^{(m-1)}\rangle$ at iteration m. We are now interested in determining the ordered d-tuple of Hermitian generators $(B_{m_d},\dots,B_{m_1})$ such that

$$
\begin{aligned}
(B_{m_d},\dots,B_{m_1}) &= \underset{B_d,\dots,B_1\in\mathbb{P}}{\operatorname{argmin}} \min_{\substack{\theta_d,\dots\theta_1\\ \in[-\pi,\pi)}} \mathscr{L}\Big((B_d,\theta_d),\dots,(B_1,\theta_1),|\Psi^{(m-1)}\rangle\Big) \\
&:= \underset{B_d,\dots,B_1\in\mathbb{P}}{\operatorname{argmin}} \min_{\substack{\theta_d,\dots\theta_1\\ \in[-\pi,\pi)}} \langle\Psi^{(m-1)}|\exp(\imath\theta_1 B_1)\dots\exp(\imath\theta_d B_d) H \exp(-\imath\theta_d B_d)\dots\exp(-\imath\theta_1 B_1)|\Psi^{(m-1)}\rangle\,.
\end{aligned}
\tag{8}
$$

In order to obtain an analytical representation of the d-dimensional objective function $\mathscr{L}\Big((B_d,\theta_d),\dots,(B_1,\theta_1),|\Psi^{(m-1)}\rangle\Big)$, the fundamental idea is to appeal once again to Equations (13) and (14) from the main text and expand each exponential in $\theta_j,\ j\in\{1,\dots,d\}$ as a sum of a sine and cosine function of θ_j. This expansion allows us to conclude that the d-dimensional objective function $\mathscr{L}\Big((B_d,\theta_d),\dots,(B_1,\theta_1),|\Psi^{(m-1)}\rangle\Big)$ can be written in the general form

$$
\mathscr{L}\Big((B_d,\theta_d),\dots,(B_1,\theta_1),|\Psi^{(m-1)}\rangle\Big) =
$$
$$
\begin{cases}
\left\langle \Psi^{(m-1)}\Big|\prod_{j=1}^{j=d}\Big(I+(\cos(\theta_j)-1)B_j^2+\imath\sin(\theta_j)B_j\Big) H \prod_{j=d}^{j=1}\Big(I+(\cos(\theta_j)-1)B_j^2-\imath\sin(\theta_j)B_j\Big)\Big|\Psi^{(m-1)}\right\rangle & \text{if } B^3=B,\\[2ex]
\left\langle \Psi^{(m-1)}\Big|\prod_{j=1}^{j=d}(\cos(\theta_j)I+\imath\sin(\theta_j)B_j) H \prod_{j=d}^{j=1}(\cos(\theta_j)I-\imath\sin(\theta_j)B_j)\Big|\Psi^{(m-1)}\right\rangle & \text{if } B^2=I.
\end{cases}
$$

In other words, the d-dimensional objective function $\mathscr{L}\Big((B_d,\theta_d),\dots,(B_1,\theta_1),|\Psi^{(m-1)}\rangle\Big)$ can be written as a polynomial of the variables $\big\{1,\cos(\theta_j),\sin(\theta_j)\colon\ j\in\{1,\dots,d\}\big\}$ with the exact structure of the polynomial depending on the properties of the operator pool $\mathbb{P}$. As a representative example, the landscape function for hardware efficient pools with $d=2$, after some simplifications, is of the form:

$$
\begin{aligned}
\mathscr{L}\big((B_2,\theta_2),(B_1,\theta_1),|\phi\rangle\big) = &\langle\phi|H|\phi\rangle \\
&+\frac{\cos(2\theta_1)}{2}\Big(\langle\phi|H-B_1HB_1|\phi\rangle+\frac{\cos(2\theta_2)}{2}\langle\phi|H-B_1HB_1-B_2HB_2+B_2B_1HB_1B_2|\phi\rangle \\
&\qquad+\frac{\sin(2\theta_2)}{2}\langle\phi|i[B_2,H-B_1HB_1]|\phi\rangle\Big) \\
&+\frac{\sin(2\theta_1)}{2}\Big(\langle\phi|i[B_1,H]|\phi\rangle+\frac{\cos(2\theta_2)}{2}\langle\phi|i[B_1,H]-iB_2[B_1,H]B_2|\phi\rangle \\
&\qquad-\frac{\sin(2\theta_2)}{2}\langle\phi|[B_2,[B_1,H]]|\phi\rangle\Big)
\end{aligned}
\tag{9}
$$

Consequently, a total of 7 observable evaluations on a quantum device are required to deduce an analytical expression for the two-dimensional landscape function $\mathscr{L}\big((B_2,\theta_2),(B_1,\theta_1),|\phi\rangle\big)$ for any Hermitian generators B_1,B_2 belonging to either of the two hardware efficient operator pools. Since the selection of the best two operator to append to the current ansatz wave-function requires comparing all pairs of Hermitian generators, we conclude that for an operator pool of size M, at most $6M^2+1$ measurements are required to determine the locally optimal pair of unitary operators that should be appended to the current ansatz wave-function at each iteration in order to achieve the largest drop in expectation value of the underlying Hermitian operator.

In the case of a general d-dimensional objective function $\mathscr{L}\Big((B_d,\theta_d),\dots,(B_1,\theta_1),|\Psi^{(m-1)}\rangle\Big)$, similar arguments yield that

- for the Qubit-Excitation-Based (QEB) operator pool of size M, we require $\mathscr{O}(5^dM^d)$ measurements to determine the locally optimal d-tuple of unitary operators that should be appended to the current ansatz wave-function at each iteration in order to achieve the largest drop in expectation value of the underlying Hermitian operator;

- for the Qubit hardware-efficient and minimal hardware-efficient pools of size M, we require $\mathcal{O}(3^d M^d)$ measurements to determine the locally optimal d-tuple of unitary operators that should be appended to the current ansatz wave-function at each iteration in order to achieve the largest drop in expectation value of the underlying Hermitian operator.

While this procedure can become computationally intractable for moderately large d, various simplifications are possible that can lead to more tractable gradient-free adaptive algorithms involving multi-operator selection and optimization, and it is likely that such methods offer an advantage when formulating a greedy gradient-free adaptive VQE for a complex Hamiltonian using a non-commutative operator pool. As representative examples, given an ansatz wave-function $|\Psi^{(m-1)}\rangle$, at iteration m:

1. We may use the energy sorting algorithm based on one-dimensional landscape functions to classify, in descending order of importance, the best d Hermitian generators $(B_d,\dots,B_1)$ whose addition to the current ansatz wave-function can result in the largest drops in the expectation value of the underlying Hermitian operator, i.e.,

$$\min_{\theta_d\in[-\pi,\pi)} \langle\Psi^{(m-1)}|\exp(\imath\theta B_d)H\exp(-\imath\theta B_d)|\Psi^{(m-1)}\rangle \geq \ldots \geq \min_{\theta_1\in[-\pi,\pi)} \langle\Psi^{(m-1)}|\exp(\imath\theta B_1)H\exp(-\imath\theta B_1)|\Psi^{(m-1)}\rangle\,.$$

   We can then switch to the analytical expression of d-dimensional landscape function $\mathcal{L}\Big((B_d,\theta_d),\dots,(B_1,\theta_1),|\Psi^{(m-1)}\rangle\Big)$ in order to compute the optimal parameters $(\theta_d^*,\dots,\theta_1^*)$ such that

$$\begin{aligned}(\theta_d^*,\dots,\theta_1^*) &= \underset{\substack{\theta_d,\dots\theta_1\\ \in[-\pi,\pi)}}{\operatorname{argmin}}\ \mathcal{L}\Big((B_d,\theta_d),\dots,(B_1,\theta_1),|\Psi^{(m-1)}\rangle\Big)\\ &= \underset{\substack{\theta_d,\dots\theta_1\\ \in[-\pi,\pi)}}{\operatorname{argmin}}\ \langle\Psi^{(m-1)}|\exp(\imath\theta_1 B_1)\dots\exp(\imath\theta_d B_d)H\exp(-\imath\theta_d B_d)\dots\exp(-\imath\theta_1 B_1)|\Psi^{(m-1)}\rangle\end{aligned}$$

   In other words, we may use one-dimensional landscape functions to identity the best d Hermitian generators to add to the current ansatz wave-function and we may employ the d-dimensional landscape functions to perform the analytical optimization. The number of quantum measurements required by this procedure scales as $3^d M$ (resp. $5^d M$) for a hardware efficient (resp. QEB) operator pool of size M.

2. Taking the newly obtained ansatz wave-function $|\Psi^{(m)}\rangle$ after iteration m as structurally fixed, we may perform Rotoselect-style[4] backwards and forwards sequential optimization sweeps over all parameterized unitary operators $\exp(\imath\theta_1^* B_d),\dots\exp(\imath\theta_d^* B_1)$. In particular, thanks to the analytical expression (9) for the d-dimensional landscape function, each iteration in these optimization sweeps can involve d parametrized unitary operators simultaneously. The number of quantum measurements required by a single sweep utilizing d-dimensional landscape functions scales as $3^d M$ (resp. $5^d M$) for a hardware efficient (resp. QEB) operator pool of size M. Algorithm 1 below (c.f., the RotoSolve algorithm[4]) yields a simple example of such a procedure for the case $d = 1$.

Numerical testing of Algorithm 1 indicates that it may alleviate– in some cases– the issue of the GGA-VQE ansatz converging to a shallow local minimum. We refer, e.g., to Figure 3 in the main text, which displays results for a stretched, linear H_6 chain for which the GGA-VQE local optimization ansatz performs poorly. However, for weakly correlated systems, the benefit of sequential re-optimization in the presence of measurement noise is less clear. Given the additional measurement overhead needed to perform re-optimization—equivalent to two or four energy evaluations per operator in the ansatz (depending on the operator type)—re-optimization becomes comparable in cost to an operator selection step only when the ansatz size approaches that of the full operator pool. For this reason, we feel that this is best treated as a post-processing step to be performed only if resources allow.

Algorithm 1 Sequential Re-optimization of GGA-VQE Ansatz

Require: At iteration m, we are given an ansatz $|\Psi^{(m)}\rangle = \exp(-\imath\widetilde{\theta_m}B_m)\dots\exp(-\imath\widetilde{\theta}_1 B_1)|\Psi^{(0)}\rangle$.

Initialize j=m-1.

while $j \geq 1$ **do**

(i) Using measurements on the quantum device, **construct** the one-dimensional landscape function

$$\mathscr{L}_j(\theta) := \Big\langle \Psi^{(0)} \Big| \exp(\imath\widetilde{\theta}_1 B_1)\dots\exp(\imath\widetilde{\theta_{j-1}}B_{j-1})\exp(\imath\theta B_j)\exp(\imath\widehat{\theta_{j+1}}B_{j+1})\dots\exp(\imath\widehat{\theta_m}B_m) \\ H\exp(-\imath\widehat{\theta_m}B_m)\dots\exp(-\imath\widehat{\theta_{j+1}}B_{j+1})\exp(-\imath\theta B_j)\exp(-\imath\widetilde{\theta_{j-1}}B_{j-1})\dots\exp(-\imath\widetilde{\theta}_1 B_1)\Big|\Psi^{(0}\Big\rangle$$

(ii) **Solve** the minimization problem

$$\widehat{\theta}_j := \underset{\theta\in[-\pi,\pi)}{\arg\min}\, \mathscr{L}_j(\theta),$$

(iii) **Update** $\widetilde{\theta}_j \mapsto \widehat{\theta}_j$ and **update** $j \mapsto j-1$.

end while

while $j \leq m$ **do**

(i) Using measurements on the quantum device, **construct** the one-dimensional landscape function

$$\mathscr{L}_j(\theta) := \Big\langle \Psi^{(0)} \Big| \exp(\imath\widehat{\theta}_1 B_1)\dots\exp(\imath\widehat{\theta_{j-1}}B_{j-1})\exp(\imath\theta B_j)\exp(\imath\widetilde{\theta_{j+1}}B_{j+1})\dots\exp(\imath\widetilde{\theta_m}B_m) \\ H\exp(-\imath\widetilde{\theta_m}B_m)\dots\exp(-\imath\widetilde{\theta_{j+1}}B_{j+1})\exp(-\imath\theta B_j)\exp(-\imath\widehat{\theta_{j-1}}B_{j-1})\dots\exp(-\imath\widehat{\theta}_1 B_1)\Big|\Psi^{(0}\Big\rangle$$

(ii) **Solve** the minimization problem

$$\theta_j := \underset{\theta\in[-\pi,\pi)}{\arg\min}\, \mathscr{L}_j(\theta),$$

(iii) **Update** $\widehat{\theta}_j \mapsto \theta_j$ and **update** $j \mapsto j+1$.

end while

6 Additional Comparison Tests for GGA-VQE and ADAPT-VQE

An additional question worth exploring is how the GGA operator selection and gradient-free analytic optimization strategy compares with the gradient-based approaches used in ADAPT-VQE. To answer this question, we consider the weakly-correlated H_2O and LiH molecules previously studied in Section 2.1., and apply a so-called "Frozen-ADAPT-VQE" algorithm to approximate their ground states. The "Frozen-ADAPT-VQE" method is a simplified version of the classical ADAPT-VQE in which the global optimization step (see, e.g., Equation (6) in Section 4.1) is replaced with a local optimization step in which only the most recently appended parameterized unitary is optimized (using an iterative algorithm). The results of this numerical test– in the same statistical noise framework considered in Section 2.1– are displayed in Figure 3. The convergence results demonstrate that, even in the noiseless regime, the GGA operator selection procedure is more optimal than the ADAPT gradient-based selection criterion and produces a modestly improved ground state. On the other hand, in the presence of statistical noise due to observable measurement, we see that GGA-VQE outperforms ADAPT-VQE thus demonstrating the noise-resilience of our approach. Note that Frozen-ADAPT-VQE outperforms standard ADAPT-VQE simulations displayed in Figure 2 in the main text, in the explored noise regime for LiH, despite sharing the same operator selection criterion. This is likely due to the fact that global optimization in the presence of measurement (statistical) noise guides the wave-function to a plateau not reached by local optimization of the last added operator.

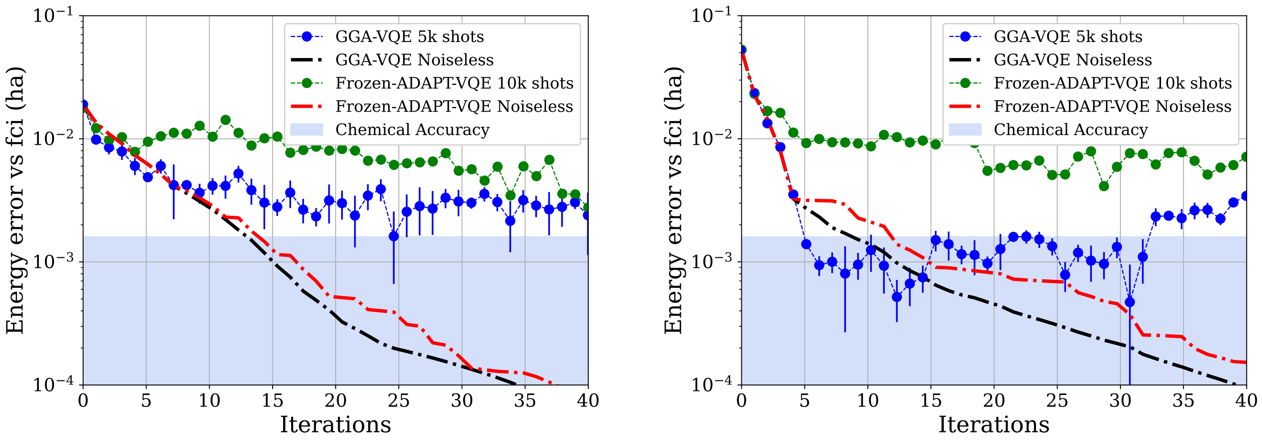

(a) H_2O **(b)** LiH

Figure 3. Comparison of the GGA-VQE algorithm with the Frozen-ADAPT-VQE algorithm, where only the last parameter is optimized and the core ansatz is kept frozen, for the ground state energy of H_2O and LiH. The plots represents the energy convergence as a function of the number of iterations of the algorithms. The shaded blue region indicates chemical accuracy at 10^{-3} Hartree. The numerical parameters used for these experiments were identical to those used for the tests in Section 2.1.

To evaluate the suitability of different optimization methods for noisy simulations, we carried out our three characteristic simulations (ground state energy of LiH, H_6 and H_2O) using BFGS as the VQE subroutine optimizer within ADAPT-VQE, both with and without shot noise. As shown in Figure 13, BFGS failed to achieve energy improvements under noisy conditions, making it unsuitable as an optimizer for this study and prompting the choice of COBYLA instead.

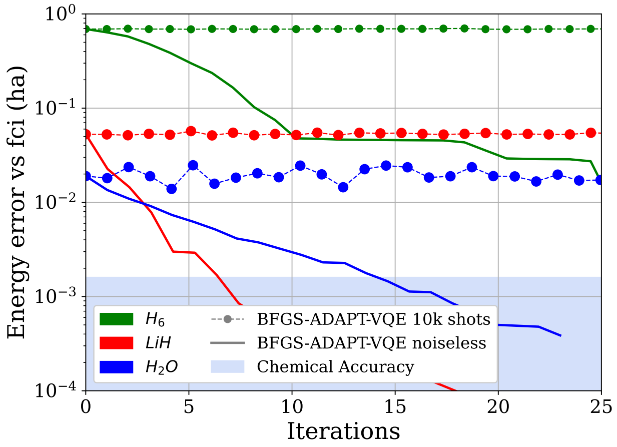

Figure 4. Comparison of ADAPT-VQE algorithm using the BFGS optimizer on the VQE subroutine under noiseless and shot noise conditions. The plots represents the energy convergence of LiH, H_6 and H_2O systems, as a function of the number of iterations of the algorithms. The shaded blue region indicates chemical accuracy at 10^{-3} Hartree.

References

1. Yordanov, Y. S., Arvidsson-Shukur, D. R. M. & Barnes, C. H. W. Efficient quantum circuits for quantum computational chemistry. Phys. Rev. A **102**, 062612 (2020).
2. Yordanov, Y. S., Armaos, V., Barnes, C. H. & Arvidsson-Shukur, D. R. Qubit-excitation-based adaptive variational quantum eigensolver. Commun. Phys. **4**, 1–11 (2021).
3. Sun, Z., Liu, J., Li, Z. & Yang, J. Circuit-efficient qubit-excitation-based variational quantum eigensolver. arXiv preprint arXiv:2406.11699 (2024).
4. Ostaszewski, M., Grant, E. & Benedetti, M. Structure optimization for parameterized quantum circuits. Quantum **5**, 391 (2021).
